# Supplementary material for: Community burden of hypertension and treatment patterns: An in-depth age predictor analysis: (The Rural Community Risk of Non-Communicable Disease Study - Nyive Phase I)
Source: PLoS One. 2021 Aug 12;16(8):e0252284. doi: 10.1371/journal.pone.0252284 (PMC8360602; doi:10.1371/journal.pone.0252284)
Supplement: S1 Table — (DOCX) [file pone.0252284.s001.docx]

**S1 Table. Treatment Options among known hypertension Residents of Nyive Community stratified by demography**

| **Parameter** | **None** | **Basic** | **Secondary** | **Tertiary** | **p value** |
| --- | --- | --- | --- | --- | --- |
| No Treatment | 9(29.03) | 42(54.55) | 10(43.48) | 3(50.00) |  |
| Hypertension Medication within 2 Weeks | 19(61.29) | 30(38.96) | 11(47.83) | 3(50.00) | 0.2102 |
| Consulted Traditional Healer on Hypertension | 19(61.29) | 25(38.96) | 7(30.43) | 2(33.33) | 0.0341 |
| Herbal Medication for Hypertension | 11(35.48) | 12(15.58) | 3(13.04) | 1(16.67) | 0.0944 |
| Herbal Medication only | 3(9.68) | 5(6.49) | 2(8.70) | 0(0.00) |  |
| Orthodox Medication only | 11(35.48) | 23(29.87) | 10(43.48) | 2(33.33) |  |
| Both Herbal & Orthodox | 8(25.81) | 7(9.09) | 1(4.35) | 1(16.67) |  |
| **Parameter** | **20 -39 years** | **40-59 years** | **60 and above** |  | **p value** |
| No Treatment | 9(81.82) | 29(41.43) | 26(46.43) |  |  |
| Hypertension Medication within 2 Weeks | 2(18.18) | 35(50.00) | 26(46.43) |  | 0.1436 |
| Consulted Traditional Healer on Hypertension | 2(18.18) | 29(41.43) | 22(46.43) |  | 0.3362 |
| Herbal Medication for Hypertension | 2(18.18) | 14(20.00) | 11(19.64) |  | 0.9900 |
| Herbal Medication only | 0(0.00%) | 6(8.57) | 4(7.14) |  |  |
| Orthodox Medication only | 0(0.00%) | 27(38.57) | 19(33.93) |  |  |
| Both Herbal & Orthodox | 2(18.18) | 8(11.43) | 7(12.50) |  |  |
| **Parameter** | **Single** | **Married** | **Divorce** | **widowed** | **p value** |
| No Treatment | 3(50.00) | 35(48.61) | 8(40.00) | 18(46.15) |  |
| Hypertension Medication within 2 Weeks | 2(33.33) | 31(43.06) | 11(55.00) | 19(48.72) | 0.704 |
| Consulted Traditional Healer on Hypertension | 2(33.33) | 29(40.28) | 10(50.00) | 12(30.77) | 0.520 |
| Herbal Medication for Hypertension | 1(16.67) | 17(23.61) | 3(15.00) | 6(15.38) | 0.689 |
| Herbal Medication only | 1(16.67) | 6(8.33) | 1(5.00) | 2(5.13) |  |
| Orthodox Medication only | 2(33.33) | 20(27.78) | 9(45.00) | 15(38.46) |  |
| Both Herbal & Orthodox | 0(0.00) | 11(15.28) | 2(10.00) | 4(10.26) |  |
| **Parameter** | **None** | **Informal** | **Formal** |  | **p value** |
| No Treatment | 2(28.57) | 60(47.62) | 2(50.00) |  |  |
| Hypertension Medication within 2 Weeks | 3(42.86) | 58(46.03) | 2(50.00) |  | 0.974 |
| Consulted Traditional Healer on Hypertension | 4(57.14) | 48(38.10) | 1(25.00) |  | 0.512 |
| Herbal Medication for Hypertension | 2(28.57) | 25(19.84) | 0(0.00) |  | 0.514 |
| Herbal Medication only | 2(28.57) | 8(6.35) | 0(0.00) |  |  |
| Orthodox Medication only | 3(42.86) | 41(32.54) | 2(50.00) |  |  |
| Both Herbal & Orthodox | 0(0.00) | 17(13.49) | 0(0.00) |  |  |
